# The action of ginsenoside Rg1 in patients with carotid atherosclerosis: a controlled clinical trial

**DOI:** 10.3389/fphar.2025.1638359

**Published:** 2025-09-02

**Authors:** Xu Fang, Xuan Ma, Man Zhang, Li Zhang, Xu He, Shilei Liu, Yinsong Dong, Yan Li, Junzi Wu

**Affiliations:** ^1^ Department of Geriatrics, The First People’s Hospital of Yunnan Province, Kunming, Yunnan, China; ^2^ The Key Laboratory of Microcosmic Syndrome Differentiation, Yunnan University of Chinese Medicine, Kunming, Yunnan, China; ^3^ Yunnan Key Laboratory of Integrated Traditional Chinese and Western Medicine for Chronic Disease in Prevention and Treatment, Yunnan University of Chinese Medicine, Kunming, Yunnan, China; ^4^ National-Local Joint Engineering Research Centre for Sanqi Resource Protection and Utilisation Technology, Kunming, Yunnan, China

**Keywords:** ginsenoside Rg1, carotid atherosclerosis, plaque, comprehensive geriatric assessment, Sanqi extract

## Abstract

**Objective:**

This study evaluated therapeutic effects of ginsenoside Rg1 (*QiShengli Tablets*) in patients with carotid artery plaques, in combination with lifestyle interventions, lipid-lowering therapy. We aimed to provide novel insights into safe clinical application of ginsenoside Rg1 for managing cardiovascular and cerebrovascular diseases.

**Methods:**

From January 2022 to October 2023, 106 carotid artery plaques patients Aged ≥60 and <74 were recruited from the Geriatrics Department of First People’s Hospital of Yunnan Province. All participants provided informed consent and a randomized sequence is generated by random number table method and divided into three groups. Group 1 received lifestyle interventions plus atorvastatin calcium tablets (n = 32). Group 2 received same treatment as Group 1, with the addition of Bayaspirin (n = 33). Group 3 received the same treatment as Group 2, but with Bayaspirin replaced by ginsenoside Rg1 (n = 41). This study adopted a single-blind design: by uniformly encapsulating the tablets in their original form in blind bags, the subjects took them at regular intervals and in fixed quantities There were no significant differences in baseline characteristics among the groups before treatment (*P* > 0.05). All patients were treated 3 months. Carotid atherosclerosis–related outcomes were assessed after treatment, and data were analyzed using SPSS 24.0.

**Results:**

Carotid ultrasound revealed significant intergroup differences in plaque number and volume changes after treatment (*P* < 0.05). No significant intergroup differences were observed in arterial stiffness index (*P* > 0.05). Fibroblast growth factor 21 levels differed significantly among the groups (*P* < 0.05), whereas Lumican and Fibulin-1 levels did not (*P* > 0.05). Analysis biochemical indicators revealed significant post-treatment differences in LDL-C, TC, Hcy, IL-6, TNF-α, 25(OH)D, and insulin resistance (IR) (*P* < 0.05). Notably, after treatment of three groups, Montreal Cognitive Assessment (MoCA) scale score was statistically significant (*P* < 0.05).

**Conclusion:**

Patients with carotid atherosclerotic plaques, adding ginsenoside Rg1 to standard therapy reduced the number and volume of carotid plaques. It improved quality of life, decreased specific inflammatory markers, and enhanced blood pressure control and Insulin Resistance (IR). These suggest ginsenoside Rg1 may have clinical value in the future management of carotid atherosclerosis.

## 1 Introduction

Cardiovascular disease (CVD) prevention and treatment remain major global challenges (Afzal et al., 2024; Jamthikar et al., 2020). According to the World Health Organization (Wang et al., 2023), approximately 120 million people worldwide suffered from CVD in 2024, accounting for 1.5% of the global population—with 60 million cases reported in China alone. CVD is a leading cause of death and disease burden, particularly among older adults. Among all CVDs, carotid atherosclerosis is of particular concern. Studies (Yuriev et al., 2021; Effati et al., 2024; Ruan et al., 2024) have shown that over 80% of individuals aged 50 and older in China have varying degrees of carotid atherosclerotic plaques. These plaques can cause arterial stenosis, reduce cerebral blood flow, and trigger ischemic cerebrovascular events, including life-threatening complications. Currently, no specific drug is approved for the treatment of carotid atherosclerosis, highlighting the urgent need for further research and therapeutic innovation.

According to clinical guidelines, the standard treatment for carotid atherosclerotic plaques involves the use of statins and Bayaspirin (López-Cancio et al., 2014; Hermel et al., 2022). Statins inhibit HMG-CoA reductase, promoting the uptake and degradation of low-density lipoprotein cholesterol (LDL-C) to lower plasma LDL-C levels (Huang et al., 2024; Turongkaravee et al., 2021; Wang et al., 2020b). Bayaspirin, an antiplatelet agent, inhibits COX-1 activity, thereby preventing abnormal platelet aggregation and reducing the risk of thrombosis (Zhang et al., 2023; Tufano et al., 2011). While this dual-drug regimen effectively slows disease progression, its long-term use is often limited by drug-related side effects, highlighting the need for safer and more sustainable alternatives (Benjamin et al., 2024; Vinci et al., 2021).

*Panax notoginseng (Sanqi)* is a well-established traditional medicine, and its saponin content, particularly ginsenoside Rg1—have become the focus of increasing scientific research (Li et al., 2008; Li et al., 2021). The Rg1-based pharmaceutical *QiShengli Tablets* (Yunnan Wenshan Teanna Pharmaceutical Co., Ltd.) have been approved for clinical use. Its core active ingredient is ginsenoside Rg1, and the formulation also includes four excipients: starch, dextrin, sucrose, and magnesium stearate. Meanwhile, the above four excipients will not affect the bioavailability and pharmacodynamic characteristics of the small molecule active ingredients within the conventional dosage range (Chen et al., 2013). Despite its approval, clinical evidence supporting its use in treating specific diseases remains limited. Consequently, the drug’s labeling describes its applications only in traditional Chinese medicine (TCM) terms—such as “invigorating the spleen,” “awakening the brain,” “reinforcing vital qi,” and “promoting blood circulation.” The lack of disease-specific indications hinders its widespread adoption in contemporary medical practice. Despite limited clinical evidence, preclinical studies over the past decade suggest that Rg1 can modulate immune function, alleviate obesity-related metabolic disorders, and exert protective effects against carotid and coronary atherosclerosis (Yang et al., 2022; He et al., 2022; Wang et al., 2020c). However, large-scale clinical trials are still needed to validate these findings.

Based on this context, our study examined the effects of ginsenoside Rg1 in patients with carotid atherosclerotic plaques. After 3 months of treatment, we conducted a comprehensive evaluation, including routine biochemical indicators (complete blood count, inflammatory markers, and oxidative stress markers) and carotid artery assessments using B-ultrasound. Ultimately, we aimed to contribute to the prevention of cardiovascular and cerebrovascular diseases and to explore ginsenoside Rg1 as a potential new therapeutic option for carotid atherosclerosis.

## 2 Materials and methods

### 2.1 Drugs and sources

Ginsenoside Rg1 (*QiShengli Tablets*): Each tablet contains 120 mg of formulation, including 15 mg of ginsenoside Rg1. Approval No.: National Drug Approval Z20027165. Manufacturer: Yunnan Teanna Pharmaceutical Co., Ltd. Atorvastatin Calcium Tablets (Lipitor): Each tablet contains 20 mg of atorvastatin calcium. Approval No.: National Drug Approval J20120049. Manufacturer: Pfizer Pharmaceuticals Ltd. Bayaspirin: Each tablet contains 100 mg of aspirin. Approval No.: National Drug Approval HJ20160685. Manufacturer: Bayer Healthcare Company Ltd.

### 2.2 Study subjects

From January 2022 to October 2023, 106 patients diagnosed with carotid atherosclerosis were enrolled from both outpatient and inpatient services at the Geriatrics Department of the First People’s Hospital of Yunnan Province. All participants provided written informed consent. Ethical approval for this study was granted in January 2023 by the Ethics Committee of the First People’s Hospital of Yunnan Province (Approval No.: KHLL2023-KY006).

#### 2.2.1 Diagnostic criteria

Patients were eligible if they met both of the following criteria:

1. Carotid plaque diagnosis was based on the “Guidelines for Vascular Ultrasound Examination” formulated by the Ultrasound Physicians Branch of the Chinese Medical Doctor Association: Normal: Intima-media thickness (IMT) < 1.0 mm. Carotid intimal thickening/atherosclerosis plaque: IMT between 1.0 mm and 1.5 mm indicates thickening. IMT ≥1.5 mm indicates plaque formation.
2. Dyslipidemia, defined by any of the following: total cholesterol (TC) > 6.2 mmol/L; triglycerides (TG) > 2.3 mmol/L; LDL-C > 4.1 mmol/L; high-density lipoprotein cholesterol (HDL-C) <1.0 mmol/L. All enrolled patients had LDL-C > 4.1 mmol/L, with concurrent use of metformin.

#### 2.2.2 Inclusion criteria

Patients were included if they met all of the following conditions:

1) Age ≥60 years old and <74 years old; 2) diagnosis of dyslipidemia; 3) presence of carotid plaque confirmed by ultrasound (IMT >1.5 mm); 4) provision of written informed consent for voluntary participation.

#### 2.2.3 Exclusion criteria

Patients were excluded if they met any of the following criteria:

1) Failed to meet the diagnostic or inclusion criteria; 2) had severe hepatic, renal, or hematologic disease; 3) were nonadherent to medication protocol; 4) had experienced recent major trauma or undergone surgery; 5) participated in another clinical trial within the past 3 months; 6) had incomplete medical records that could not be supplemented.

### 2.3 Treatment and grouping

One hundred six patients were randomized into three groups. Group 1 (n = 32): received a combination of lifestyle intervention and atorvastatin calcium tablets. Group 2 (n = 33) received the same treatment as Group 1, with the addition of Bayaspirin enteric-coated tablets. Group 3 (n = 41) received the same treatment as Group 2, but Bayaspirin was discontinued and replaced with ginsenoside Rg1 (*QiShengli Tablets*). The lifestyle intervention implemented across all groups consisted of several components. Patients were advised to adopt reasonable dietary and lifestyle modifications, including a low-salt, low-fat diet; cessation of smoking; limited alcohol consumption; and increased intake of vegetables, lean protein, and fish. A small quantity of nuts was allowed daily, while the intake of fructose was to be minimized. Specific dietary targets included restricting daily cholesterol intake to less than 300 mg, maintaining a daily fiber intake of 25–30 g per day, and limiting salt consumption to below 5 g per day. In addition, patients were encouraged to get adequate rest, maintain a positive outlook, and engage in regular physical activity. They were permitted to select their preferred exercise methods, such as brisk walking, jogging, aerobics, or swimming. Each exercise session lasted 45 min and was performed five times per week over a 3-month period. Pharmacological treatment for Group 1 included atorvastatin calcium tablets administered at a dose of 20 mg once nightly. In Group 2, Bayaspirin enteric-coated tablets were added to the regimen (100 mg taken once every morning). In Group 3, Bayaspirin was discontinued, and patients instead received ginsenoside Rg1 (two QiShengi Tablets taken three times daily over a 3-month period). Relevant data were collected after the 3-month treatment period, and subsequent related experiments were conducted.

Before the study, *a priori* power analysis was conducted using G*Power software to determine the required sample size. Based on the anticipated effect size, a two-tailed α of 0.05, and a target power of at least 50%, the minimum required sample size was 32 participants per group. The final sample sizes met or exceeded this threshold, ensuring sufficient statistical power to detect significant differences between the groups. Throughout the study period, no participants withdrew or dropped out, and all enrolled patients adhered strictly to the study protocol.

### 2.4 Data collection

A single-anonymized approach was adopted for this study. Tablets from different batches were repackaged into medication bags for patients to take daily. Tablet crushing was not performed, as Bayaspirin is a sustained-release formulation. The ethics committee raised concerns that crushing the tablets could compromise drug efficacy and, hence, did not approve this approach. Additionally, to ensure the accuracy and objectivity of the data collection, all clinical and laboratory data were collected by an independent physician who was blinded to the group assignments. The physician primarily assessed the following indicators: 1) General patient characteristics: This included gender and the presence of vascular risk factors for carotid artery plaques, including hypertension, diabetes, smoking history, and alcohol consumption. Patients with these risk factors were required to have stable control of blood pressure and blood glucose for at least 6 months prior to enrollment, with no need for medical adjustments during the study period. Moreover, Patients with chronic conditions continued their baseline treatments throughout the study to maintain blood pressure and glucose control. 2) Laboratory tests: Blood samples were uniformly processed at the Biochemistry Laboratory of the Laboratory Department, First People’s Hospital of Yunnan Province, using an automatic biochemical analyzer. The following markers were assessed: complete blood count, liver and kidney function, lipid profile, glycated hemoglobin, fasting blood glucose, fasting insulin, uric acid, 25(OH)D, and pro-inflammatory markers (e.g., CRP, IL-10, IL-2, IL-4, IL-6, TNF-α, and IFN-γ). Oxidative stress markers included MDA, ROS, SOD, and GSH. 3) Carotid ultrasound: Carotid plaque location and volume were evaluated using B-mode ultrasound. Plaque volume was estimated using standard imaging protocols. 4) Pulse wave velocity (PWV) and ankle-brachial index (ABI) were measured to evaluate arterial stiffness. 5) Vascular aging indicators: Levels of Lumican, fibroblast growth factor 21 (FGF21), and Fibulin-1 were tested at the Yunnan Key Laboratory of TCM Micro-Dialectics. 6) Comprehensive geriatric assessments: Cognitive and psychological status were evaluated using the Montreal Cognitive Assessment (MoCA), Hamilton Anxiety Rating Scale. (HAMA), Pittsburgh Sleep Quality Index (PSQI), and Hamilton Depression Scale (HAMD).

### 2.5 Statistical analysis

Data were analyzed using SPSS version 24.0 (IBM SPSS, United States). Categorical variables were expressed as percentages (%) and analyzed using the χ^2^ test. Continuous variables are presented as mean ± standard deviation. Differences among the three groups were assessed using a one-way analysis of variance, while differences before and after treatment within each group were evaluated using paired sample *t*-tests. A two-tailed *p*-value of <0.05 was considered statistically significant for all comparisons.

### 2.6 Technical roadmap

**FIGURE 1:**
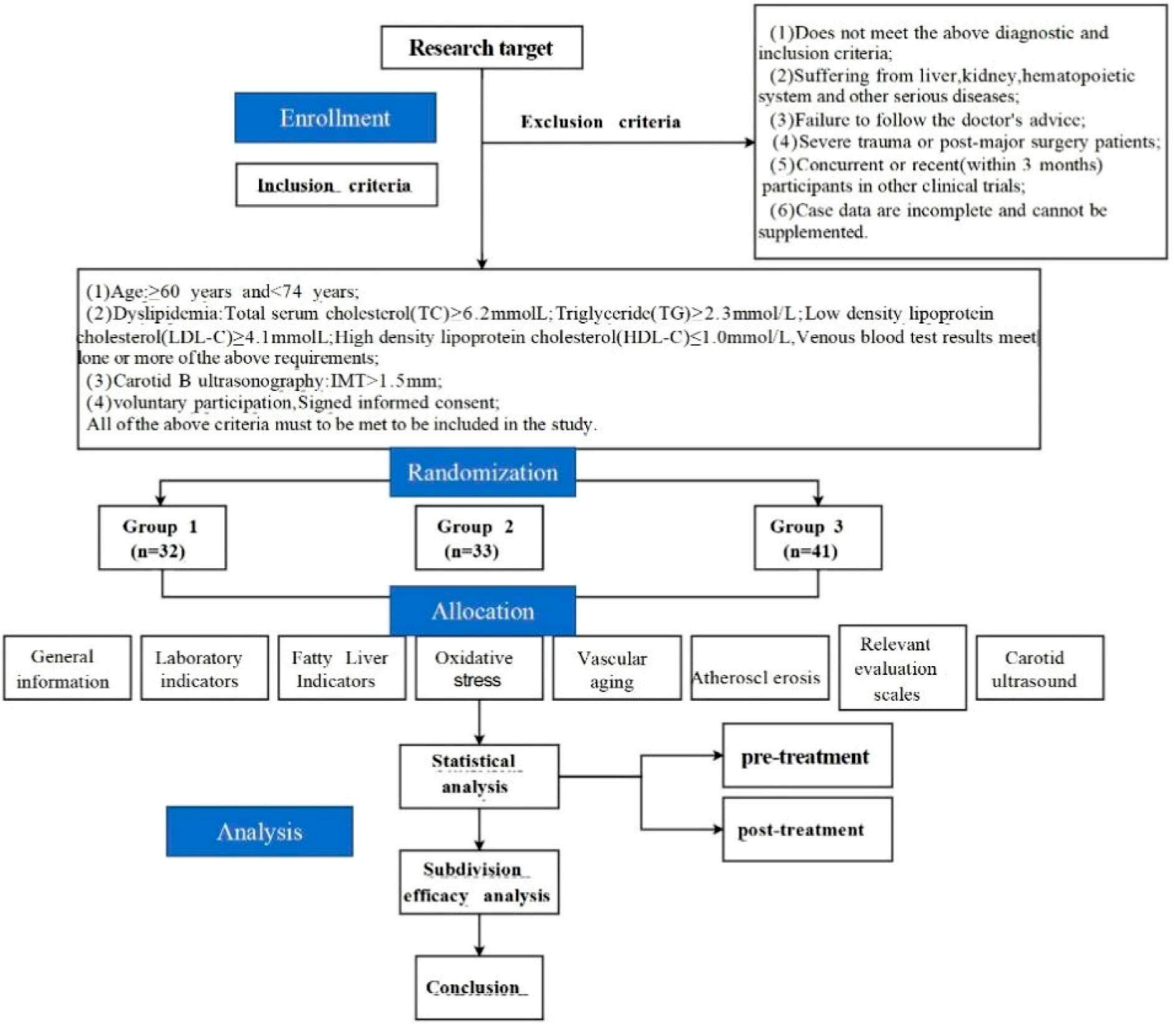
Technical route. (Note: This study strictly adhered to the treatment plan. All enrolled patients followed the advice throughout the process, taking the study drugs on time and in the prescribed dosage. There were no cases of poor medication compliance or drug withdrawal during the study, no dropout, ensuring the integrity and reliability of the research data).

The experimental design and workflow of this study are illustrated in Figure 1.

## 3 Results

### 3.1 Baseline characteristics

Before treatment, no significant differences were observed among the three groups in terms of gender, age, hypertension, diabetes mellitus, smoking history, or alcohol consumption (see Table 1 for details). Additionally, baseline laboratory and imaging markers—including blood tests (complete blood count), serum inflammatory cytokines, and carotid atherosclerosis indicators (e.g., IMT and plaque volume)—were comparable across the groups (Supplementary Tables S1–S6).

**TABLE 1:** Comparison of the basic conditions of the two groups of patients.

Group/Indicator		Group 1	Group 2	Group 3	P
Gender	Male	15 (48.39%)	16 (48.48%)	22 (53.66%)	0.91
	Female	16 (51.61%)	17 (51.51%)	19 (46.34%)	
Age	60∼65	13 (48.39%)	15 (48.48%)	25 (53.66%)	0.35
	66∼70	12 (51.61%)	9 (51.51%)	12 (46.34%)	
	70∼74	6 ()	9 ()	4 ()	
Hypertension	Having	13 (41.94%)	9 (27.27%)	20 (48.78%)	0.80
	Not having	18 (58.06%)	24 (72.73%)	21 (51.22%)	
Diabetes	Having	4 (12.90%)	5 (15.15%)	9 (21.95%)	0.94
	Not having	27 (87.10%)	28 (84.85%)	32 (78.05%)	
Smoke	Having	8 (25.81%)	7 (21.21%)	9 (21.95%)	0.92
	Not having	23 (74.19%)	26 (78.79%)	32 (78.05%)	
Drinking	Having	5 (16.13%)	9 (27.27%)	9 (21.95%)	0.61
	Not having	26 (83.87%)	24 (72.73%)	32 (78.05%)	

### 3.2 Carotid atherosclerosis indicators

#### 3.2.1 Carotid ultrasound parameters

Plaque Quantity Analysis: There were no statistically significant differences in plaque quantity among the three groups before treatment (Supplementary Table S5). Carotid ultrasound was used to assess changes within each group (pre-vs. post-treatment) and between groups following treatment (Table 2).

**TABLE 2:** Comparison of the number of plaque locations before and after treatment among the three groups.

Plaque location (number)		Pre-treatment	Post-treatment	P_1_	P_2_
Group 1	Left	16	15	0.873	0.019*
	Right	38	40		
Group 2	Left	18	7	0.035*	
	Right	33	16		
Group 3	Left	23	8	0.024*	
	Right	34	22		

Note: Left side numbers include the number of plaques on the left side of the common carotid artery, left side of the subclavian artery, left side of the internal carotid artery, and left side of the external carotid artery; Right-sided numbers include the number of plaques on the right side of the common carotid artery, the right side of the subclavian artery, the right side of the internal carotid artery, and the right side of the external carotid artery. Note:*P < 0.05; P1 is the before and after control within the group itself, P2 is the comparison of the number of plaque locations between-group control after treatment. The overall trend of efficacy after treatment was better in group 3 than group 2 than group 1.

Group 1: Left carotid plaques decreased, while right carotid plaques increased slightly; however, neither change was statistically significant (*P* > 0.05). Group 2: A significant reduction was observed in both left and right carotid plaques after treatment (*P* < 0.05). Group 3: Similar to Group 2, a significant reduction was observed in both left and right carotid plaques (*P* < 0.05). Intergroup comparison revealed statistically significant differences in post-treatment plaque quantity among the three groups (*P* < 0.05), indicating varied responses to the interventions.

**FIGURE 2:**
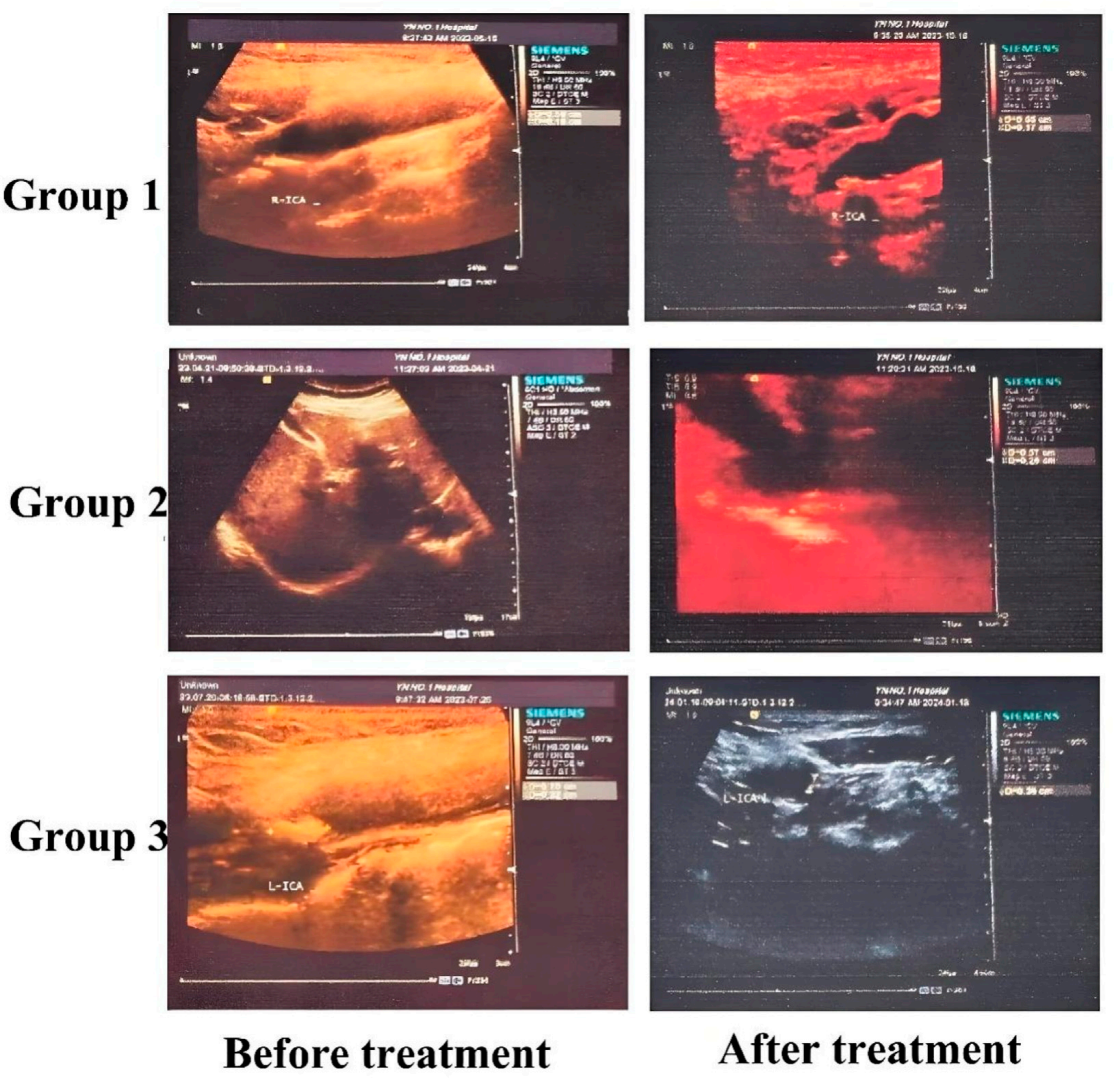
Ultrasound results graph of some patients.

The characteristics of carotid plaque volume changes are presented in Table 3. After treatment, Groups 1 and 2 both showed reductions in plaque volume; however, these changes were not statistically significant (*P* > 0.05). In contrast, Group 3 exhibited a significant reduction in plaque volume (*P* < 0.05). Post-treatment intergroup analysis also revealed statistically significant differences in plaque volume among the three groups (*P* < 0.05). Overall, Group 3 showed the most pronounced therapeutic effect, suggesting that a combination of lifestyle intervention, atorvastatin therapy, and intensified exercise can contribute to carotid plaque reduction. Furthermore, replacing Bayaspirin with ginsenoside Rg1 in this regimen appeared to be more effective in reducing carotid volume. The detailed plaque volume measurements are presented in Table 3. In addition, we selected some representative B-ultrasound images, See Figure 2, with additional diagnostic ultrasound results provided in Supplementary Figures S1–S3. From the figure, the carotid plaque volumes before treatment were as follows: Group 1 had a plaque measuring 5.9 × 4.0 × 1.8 mm; Group 2 had a plaque measuring 7.7 × 5.9 × 2.3 mm; and Group 3 had two plaques, measuring 4.2 × 4.1 × 2.6 mm and 7.0 × 3.4 × 3.4 mm. After 3 months of treatment, the corresponding plaque volumes were reduced to 6.5 × 3.6 × 1.7 mm in Group 1, 5.7 × 5.2 × 2.6 mm in Group 2, and 3.3 × 3.5 × 1.5 mm and 7.0 × 3.0 × 2.0 mm in Group 3. Although these measurements showed varying degrees of plaque reduction across all groups, the most pronounced decrease was observed in Group 3.

**TABLE 3:** Comparison of plaque volume before and after treatment among the three groups.

Plaque volume	Pre-treatment	Post-treatment	P_1_	P_2_
Group 1	36.480 (15.9,94.6)	34.230 (20.8,61.5)	0.073	
Group 2	25.578 (11.8,69.5)	21.578 (14.5,63.1)	0.119	0.021*
Group 3	44.280 (25.4,65.1)	31.960 (11.1,73.2)	0.005*	

Note: *P < 0.05; **P < 0.01; P1 is pre-treatment compared with post-treatment for the corresponding group; P2 is the comparison of plaque volume post-treatment for the three groups. The overall trend of efficacy after treatment was better in group 3 than group 2 than group 1.

#### 3.2.2 Arterial stiffness indices

Atherosclerosis indices before and after treatment were comparable across all three groups, as detailed in Supplementary Table S4. Analysis of arterial stiffness indicators revealed the following key findings: statistically significant improvements in PWV at the right ankle-brachial site were observed in Groups 1 and 3 (*P* < 0.05). A statistical change in the left ankle-brachial PWV was observed only in Group 3 (*P* < 0.05). No other arterial stiffness indices showed significant changes in any of the groups (*P* > 0.05). For the between-group comparison, no statistically significant differences in arterial stiffness indicators were found among the three groups post-treatment (*P* > 0.05). These results suggest a potential role for lifestyle intervention, atorvastatin calcium therapy, intensive exercise, and ginsenoside Rg1 in improving arterial elasticity. Detailed results are presented in Table 4.

**TABLE 4:** Comparison of atherosclerosis indexes before and after treatment in three groups.

Project	Groups	Pre-treatment	Post-treatment	P_1_	P_2_
Right Ankle-Brachial Index (ABI)	Group 1	1.160 (1.1,1.3)	1.109 (1.0,1.2)	0.568	0.071
	Group 2	1.198 (1.1,1.3)	1.188 (1.1,1.3)	0.362	
	Group 3	1.237 (1.1,1.3)	1.186 (1.0,1.4)	0.023*	
Right Ankle-Brachial PWV	Group 1	1621.00 (1502.0,1735.0)	1640.000 (1381.0,1752.0)	0.725	0.448
	Group 2	1691.00 (1446.0,1958.5)	1667.000 (1359.0,1827.0)	0.602	
	Group 3	1598.00 (1402.5,1725.5)	1544.000 (1323.5,1649.5)	0.006*	
Left Ankle-Brachial Index (ABI)	Group 1	1.178 (1.1,1.3)	1.091 (1.0,1.2)	0.071	0.375
	Group 2	1.151 (1.1,1.3)	1.154 (1.0,1.3)	0.448	
	Group 3	1.193 (1.1,1.3)	1.172 (1.0,1.4)	0.375	
Left Ankle-Brachial PWV	Group 1	1695.61 ± 221.87	1671.48 ± 257.48	0.746	0.448
	Group 2	1796.33 ± 323.81	1639.27 ± 308.78	0.423	
	Group 3	1691.90 ± 301.75	1593.85 ± 257.21	0.039*	

Note: *P < 0.05; **P < 0.01; P1 is pre-treatment compared with post-treatment for the corresponding group; P2 is the comparison of atherosclerosis indexes post-treatment for the three groups.

#### 3.2.3 Vascular senescence indexes

There were no significant differences in vascular senescence indices among the three groups before treatment (Supplementary Table S3). Vascular aging indicators were analyzed within each group (pre-vs. post-treatment) and compared across groups after treatment, with results summarized in Table 5. In Group 1, there were no statistically significant changes in Lumican, FGF21, or Fibulin-1 levels after treatment (*P* > 0.05). Similarly, in Group 2, no statistically significant differences were found in these three markers after (*P* > 0.05). However, in Group 3, FGF21 levels increased significantly after treatment (*P* < 0.05), while changes in Lumican and Fibulin-1 remained statistically insignificant (*P* < 0.05). Intergroup comparisons after treatment revealed a statistically significant difference in FGF21 levels among the three groups (*P* < 0.05), with no significant differences in Lumican or Fibulin-1 (*P* > 0.05). The overall trend in treatment efficacy indicated that Group 3 outperformed Group 2, which in turn outperformed Group 1. These findings suggest that ginsenoside Rg1 may enhance the improvement of vascular senescence when added to a regimen consisting of lifestyle intervention, atorvastatin calcium therapy, and intensive exercise.

**TABLE 5:** Comparison of therapeutic antioxidant indexes among the three groups.

Project	Groups	Pre-treatment	Post-treatment	P_1_	P_2_
Lumican	Group 1	31.550 (30.4,34.4)	30.200 (6.1,34.6)	0.306	0.071
	Group 2	31.770 (30.4,33.6)	33.040 (7.5,34.4)	0.239	
	Group 3	31.450 (30.1,34.0)	33.340 (8.3,41.5)	0.247	
FGF21	Group 1	1305.730 (1228.1,1432.3)	1368.860 (1179.5,1481.5)	0.372	0.000**
	Group 2	1300.950 (1211.5,1411.3)	1380.290 (1274.1,1451.1)	0.286	
	Group 3	1311.500 (1213.6,1412.1)	1701.330 (1621.8,1756.2)	0.006**	
Fibulin1	Group 1	318.890 (301.4,345.5)	318.300 (283.4,338.2)	0.733	0.295
	Group 2	333.190 (312.1,359.6)	322.300 (292.6,336.2)	0.536	
	Group 3	328.600 (315.3,348.6)	359.130 (344.2,377.8)	0.230	

Note: *P < 0.05; **P < 0.01; P1 is pre-treatment compared with post-treatment for the corresponding group; P2 is the comparison of therapeutic antioxidant indexes post-treatment for the three groups. After treatment, regarding FGF21, the overall efficacy trend was better in group 3 than group 2 than group 1.

### 3.3 Laboratory test indicators

There were no differences in laboratory test indicators among the three groups before treatment, indicating comparable baseline values (see Supplementary Tables S1, S2). Pre- and post-treatment comparisons within each group are presented in Table 6. In Group 1, some indicators showed improvement after treatment, but the differences were not statistically significant (*P* > 0.05). In Group 2, significant improvements were observed in LDL-C, Hcy, and insulin resistance (IR, *P* < 0.05). In Group 3, statistically significant improvements were found in LDL-C, IL-6, TNF-α, Hcy, 25(OH)D, and IR (*P* < 0.05). When comparing post-treatment results among all groups, significant differences were observed in LDL-C, TC, Hcy, IL-6, TNF-α, 25(OH)D, and IR (*P* < 0.05). The trend in efficacy followed the order: Group 3 > Group 2 > Group 1. These results suggest that Bayaspirin has a certain effect on reducing LDL-C, Hcy and IR on the basis of intensified exercise, lifestyle intervention and lipid-lowering treatment with atorvastatin calcium. Additionally, the inclusion of ginsenoside Rg1 that replacing Bayaspirin further amplified these effects, demonstrating a pronounced impact on lowering LDL-C, TC, Hcy, and IR levels while increasing 25(OH)D levels.

**TABLE 6:** Comparison of relevant laboratory examination indexes before and after treatment among the three groups.

Project	Groups	Pre-treatment	Post-treatment	P_1_	P_2_
Blood lipids					
TC (mmol/L)	Group 1	5.670 (1.3,6.2)	5.630 (1.3,6.1)	0.719	0.046*
	Group 2	5.600 (1.0,6.1)	5.500 (1.1,6.1)	0.816	
	Group 3	5.560 (1.1,6.0)	5.245 (1.0,6.0)	0.981	
TG (mmol/L)	Group 1	1.670 (1.3,2.2)	1.520 (1.1,1.7)	0.313	0.937
	Group 2	1.600 (1.0,2.1)	1.510 (1.0,1.8)	0.482	
	Group3	1.560 (1.1,2.0)	1.500 (1.1,1.8)	0.083	
HDL-C (mmol/L)	Group 1	1.270 (1.1,1.5)	1.305 (1.2,1.5)	0.144	0.337
	Group 2	1.330 (1.2,1.7)	1.410 (1.2,1.8)	0.482	
	Group 3	1.430 (1.2,1.6)	1.495 (1.2,1.8)	0.083	
LDL-C (mmol/L)	Group 1	3.010 (2.3,3.6)	2.980 (2.3,3.5)	0.154	0.003**
	Group 2	2.955 (2.0,3.5)	2.580 (2.1,3.0)	0.010**	
	Group 3	3.300 (2.3,4.0)	2.160 (1.6,2.6)	0.000**	
Serum pro-inflammatory mediators					
IL-10 (pg/mL)	Group 1	3.400 (1.9,6.5)	2.245 (1.5,2.6)	0.895	0.297
	Group 2	2.860 (2.4,5.0)	2.510 (2.2,4.0)	0.295	
	Group 3	3.050 (2.6,4.3)	1.915 (1.3,3.1)	0.198	
IL-2 (pg/mL)	Group 1	1.570 (0.8,4.3)	2.820 (1.4,4.3)	0.755	0.889
	Group 2	1.700 (1.1,3.1)	2.810 (1.6,3.0)	0.684	
	Group 3	1.240 (0.6,3.3)	2.455 (1.7,3.4)	0.819	
IL-4 (pg/mL)	Group 1	1.870 (1.0,4.3)	3.530 (1.7,5.9)	0.320	0.129
	Group 2	1.690 (0.9,2.8)	2.720 (1.1,3.9)	0.802	
	Group 3	2.180 (1.0,3.5)	2.785 (2.0,4.1)	0.436	
IL-6 (pg/mL)	Group 1	6.630 (3.1,9.9)	6.130 (2.9,11.4)	0.250	0.020*
	Group 2	6.640 (2.3,12.2)	5.840 (2.8,11.6)	0.061	
	Group 3	6.720 (3.4,11.5)	5.620 (2.9,10.3)	0.002**	
TNF-α	Group 1	1.876 (0.9,4.7)	1.703 (0.7,1.8)	0.953	0.041*
	Group 2	1.560 (1.5,2.5)	1.560 (1.2,2.0)	0.563	
	Group 3	1.710 (1.0,2.9)	0.990 (0.6,1.8)	0.016*	
IFN-γ	Group 1	5.006 (2.7,9.3)	3.705 (2.2,6.1)	0.791	0.133
	Group 2	3.920 (2.3,6.7)	4.160 (2.5,6.5)	0.280	
	Group 3	4.422 (2.8,6.8)	3.885 (2.7,5.5)	0.473	
Other					
Hcy (umol/L)	Group 1	13.700 (11.2,16.8)	13.200 (11.7,16.0)	0.836	0.030*
	Group 2	13.400 (10.5,18.0)	12.000 (10.5,17.6)	0.014*	
	Group 3	13.700 (11.4,15.4)	11.500 (10.1,13.4)	0.009**	
25(OH)D	Group 1	23.370 (18.8,29.7)	24.330 (22.4,26.4)	0.215	0.005**
	Group 2	24.640 (22.7,33.9)	26.120 (23.7,30.1)	0.201	
	Group 3	26.220 (21.9,30.9)	28.900 (25.9,31.6)	0.037*	
IR	Group 1	2.672 (1.3,4.1)	2.653 (2.3,3.4)	0.102	0.014*
	Group 2	2.105 (1.3,3.8)	2.119 (1.5,2.4)	0.044*	
	Group 3	2.537 (1.1,3.7)	1.711 (1.3,2.4)	0.000**	
HbA1C	Group 1	6.010 (5.9,6.2)	6.190 (5.9,6.4)	0.625	0.159
	Group 2	6.060 (5.8,6.7)	6.170 (6.0,6.6)	0.513	
	Group 3	5.870 (5.7,6.2)	6.075 (5.8,6.4)	0.305	

Note: *P < 0.05; **P < 0.01; P1 is pre-treatment compared with post-treatment for the corresponding group; P2 is the comparison of relevant laboratory examination indexes post-treatment for the three groups. After treatment, regarding TC, LDL-C, IL-6, TNF-α, Hcy, 25(OH)D, IR, the overall efficacy trend was better in group 3 than group 2 than group 1.

### 3.4 Geriatric comprehensive assessment scale

Pre- and post-treatment comparisons were conducted within each of the three groups. According to records from the designated investigators, there were no significant differences among the three groups at baseline in terms of cognitive function, emotional state, sleep quality, or treatment compliance, indicating comparability. Before treatment, there were no statistically significant differences in the MoCA, HAMA, PSQI, and HAMD scores among the groups (see Supplementary Table S6). After treatment, statistically significant differences in MoCA scores were observed among the groups (*P* < 0.05; see Table 7). In Group 1, no statistically significant changes were observed in any of the assessment indicators after treatment (*P* > 0.05). In contrast, both Group 2 and Group 3 demonstrated statistically significant improvements in MoCA scores post-treatment compared to baseline (*P* < 0.05). Intergroup comparisons of post-treatment MoCA scores also indicated statistically significant differences (*P* < 0.05). However, no statistically significant changes were found in HAMA, PSQI, and HAMD scores across or within groups (*P* > 0.05). These results suggest that a combination of lifestyle interventions, atorvastatin calcium therapy, and intensive exercise may contribute to improved cognitive function. Furthermore, replacing Bay aspirin with ginsenoside Rg1 appears to yield a more pronounced enhancement in cognitive outcomes. However, the intervention had no significant impact on anxiety or depression levels (see Table 7).

**TABLE 7:** Comparison of scale scores before and after treatment among the three groups.

Scale score	Groups	Pre-treatment	Post-treatment	P_1_	P_2_
MoCA	Group 1	24.000 (22.0,27.0)	24.500 (23.0,28.0)	0.362	0.036*
	Group 2	25.000 (23.0,26.0)	27.000 (24.5,28.0)	0.049*	
	Group 3	23.000 (21.5,26.0)	26.000 (25.0,28.0)	0.012*	
HAMA	Group 1	6.000 (5.0,8.0)	6.000 (2.0,6.0)	0.928	0.357
	Group 2	6.000 (2.0,8.0)	4.000 (2.0,6.0)	0.602	
	Group 3	6.000 (2.0,8.5)	4.000 (1.3,6.0)	0.876	
PSQI	Group 1	5.000 (3.5,6.0)	5.000 (4.0,6.0)	0.510	0.236
	Group 2	5.000 (4.0,7.5)	6.000 (4.0,7.0)	0.253	
	Group 3	6.000 (4.0,7.4)	4.000 (2.0,5.8)	0.519	
HAMD	Group 1	6.000 (5.0,11.0)	6.000 (2.0,8.8)	0.604	0.567
	Group 2	6.000 (4.5,9.0)	6.000 (2.0,8.0)	0.782	
	Group 3	6.000 (4.0,8.5)	4.000 (2.0,6.0)	0.677	

Note: *P < 0.05; P1 is pre-treatment compared with post-treatment for the corresponding group; P2 is the comparison of scale scores post-treatment for the three groups. After treatment, regardingMoCA, the overall efficacy trend was better in group 3 than group 2 than group 1.

## 4 Discussion

As noted in the introduction, carotid atherosclerosis is a specific form of atherosclerosis primarily characterized by lesions in the carotid intima, lipid deposition, luminal narrowing, and subsequent plaque formation (Dong et al., 2022; Liu et al., 2023). Studies have identified several key risk factors for carotid atherosclerotic plaque development, including age, sex, hypertension, hyperglycemia, and levels of HDL-C and LDL-C (Tian et al., 2024; Papazoglou et al., 2024). Current clinical treatment strategies involve a combination of pharmacological interventions—such as blood pressure control, plaque stabilization, and antithrombotic therapy—and lifestyle modifications (e.g., smoking cessation, regular physical activity, and weight management). Additional goals include controlling underlying risk factors, such as diabetes, hypertension, and hyperlipidemia, often through medications like antiplatelet agents, antihypertensives, and lipid-lowering drugs (Cao et al., 2024; Yang et al., 2020; Zhang and Ma, 2022). The combination of Bayaspirin and atorvastatin has been reported to significantly slow disease progression; however, its use may be limited by adverse side effects, underscoring the need for alternative therapies. Ginsenoside Rg1, the primary active component of *Panax notoginseng* (Sanqi), has demonstrated therapeutic effects in animal models of carotid atherosclerosis. However, its clinical efficacy remains largely unexplored (Wang et al., 2020a; Gu et al., 2022; Hu et al., 2025). Preliminary findings from our team’s preclinical, early clinical observations and access to relevant literature also suggest that ginsenoside Rg1 possesses anticoagulant properties (Liu et al., 2024). Therefore, we aimed to evaluate its therapeutic value by substituting Bayaspirin with ginsenoside Rg1 and comparing the outcomes. Post-treatment outcomes were assessed after a 3-month intervention, with comprehensive comparisons made between pre- and post-treatment results and across study groups.

Carotid color Doppler ultrasound is a key diagnostic tool for vascular health evaluation. It enables the detection of intimal thickening, plaque formation, plaque size, and plaque stiffness and allows for the measurement of blood flow velocity to evaluate stenosis or occlusion (Wen et al., 2022; Saba et al., 2024; Riggin et al., 2021). This modality is particularly valuable for monitoring treatment efficacy in patients with moderate-to-severe carotid stenosis or occlusion. In our study, carotid ultrasound revealed that both exercise therapy alone and exercise combined with ginsenoside Rg1 significantly reduced the number of plaques in both the left and right arteries (*P* < 0.05). Although no significant difference was found between these two groups in terms of plaque quantity reduction, the group receiving ginsenoside Rg1 exhibited a significantly greater plaque volume than the group receiving exercise plus Bayaspirin, highlighting the superior effect of ginsenoside Rg1.

To further assess the therapeutic benefits of ginsenoside Rg1, we assessed the arterial stiffness index (ASI) and the vascular health index (VHI). ASI is closely associated with carotid atherosclerosis, as elevated values correlate with increased IMT, reflecting disease progression. The VHI offers a broader assessment of an individual’s vascular condition and lifestyle-related cardiovascular risk. Given that the carotid artery is an important conduit supplying blood from the heart to the brain, its integrity directly impacts cerebral perfusion (Csipo et al., 2025). Carotid artery lesions, such as those caused by atherosclerosis, can lead to severe outcomes like stroke. Thus, we jointly evaluated the differences between the ASI and VHI. Although no significant post-treatment differences in ASI were observed between the groups, the ginsenoside Rg1 group showed significant improvements in left and right ankle-brachial PWV. These findings support the relatively higher therapeutic potential of ginsenoside Rg1 compared to Bayaspirin. Additionally, FGF21 levels differed significantly among the groups, while Lumican and Fibulin-1 showed no statistically significant variations. This result further proves that ginsenoside Rg1 can provide additional vascular benefits in managing carotid atherosclerosis compared to Bayaspirin.

Following the aforementioned procedures, we further examined the effects of ginsenoside Rg1 on biochemical parameters in the treated patient groups. Comparative analysis revealed statistically significant intergroup differences (*P* < 0.05) in LDL-C, TC, Hcy, IL-6, TNF-α, 25(OH)D, and IR levels post-treatment. Notably, LDL-C and TC—both established risk factors for atherosclerosis—demonstrated significant reductions following ginsenoside Rg1 administration, confirming its lipid-lowering potential. Additionally, ginsenoside Rg1 significantly improved five key biomarkers: Hcy, IL-6, TNF-α, 25(OH)D, and IR. Among these, IL-6 and TNF-α are key pro-inflammatory cytokines involved in all stages of carotid atherosclerosis—from plaque formation and progression to rupture. Elevated levels of these cytokines are known to promote inflammatory responses and exacerbate vascular endothelial injury, thereby compromising plaque stability. The inflammatory reaction can promote the rupture of foam cells after phagocytosis of lipids, releasing more lipid content that accelerates the increase in the atherosclerotic core volume; Meanwhile, the inflammatory reaction can also cause carotid vascular remodeling, which leads to further narrowing of the vascular lumen (Jin et al., 2022). Moreover, low levels of 25(OH)D have been associated with hypertensive carotid atherosclerosis, and its concentration is negatively correlated with plaque severity. IR and Hcy are also related to diabetic carotid atherosclerosis, particularly IR, which has been associated with increased IMT, greater arterial stiffness, and reduced vascular compliance and dilation capacity (Sipos et al., 2021; Strisciuglio et al., 2020; Liu et al., 2019). This suggests that IR may negatively impact carotid artery health by altering the structure and function of the vascular wall. The observed improvements in these biomarkers following ginsenoside Rg1 treatment highlight its potential therapeutic value, particularly for patients with hypertension or diabetic carotid atherosclerosis.

Finally, we assessed the three patient groups before and after treatment using the Comprehensive Geriatric Assessment Scales—MoCA, HAMA, PSQI, and HAMD—to evaluate whether ginsenoside Rg1 can improve the overall quality of life in patients with carotid atherosclerosis. Among these scales, the MoCA evaluates cognitive function, the HAMA assesses anxiety levels, the PSQI measures sleep quality, and the HAMD assesses depressive symptoms. Our results indicated that after 3 months of ginsenoside Rg1 treatment, there were no significant changes in HAMA, PSQI, and HAMD scores. This suggests that ginsenoside Rg1 does not significantly improve depression, anxiety, or sleep quality in patients with carotid atherosclerosis. However, the MoCA scores showed a significant difference among the three groups, suggesting that ginsenoside Rg1 may have a positive effect on cognitive function. For some of the indicators that did not reach statistical significance, we analyzed that they might be affected by 1) sample size limitations, especially the small number of cases in some subgroup analyses; 2) heterogeneity of responses to Rg1 due to inter-individual metabolic differences; and 3) the short follow-up period of the study, where changes in some biomarkers had not yet been adequately revealed. Future studies could be further validated by expanding the sample size, extending the intervention time, or using more sensitive assays.

In summary, our clinical study demonstrates that adjunctive ginsenoside Rg1 therapy—when combined with lifestyle modifications, lipid-lowering treatment, and intensive exercise—may reduce carotid plaque volume and number while also improving quality of life and certain metabolic and inflammatory markers. However, several limitations should be acknowledged: 1) The exclusion of high-risk patients with severe carotid atherosclerosis or multiple comorbidities may have introduced selection bias. 2) All participants in this study were older adults with underlying conditions and various geriatric syndromes. Despite efforts to minimize confounding factors, some influences on the outcomes may have been unavoidable. 3) The study was cross-sectional with regional limitations; therefore, the findings may not be generalizable to the broader elderly population. 4) The intervention period was limited to 3 months, and the sample size was relatively small. Although *a priori* power analysis (G*Power) suggested a minimum of 32 participants per group for detecting a medium effect size, larger sample sizes (e.g., >180 per group) would be required for more robust conclusions. Therefore, in subsequent studies, we plan to expand the sample size and extend the observation period to clarify the therapeutic potential of ginsenoside Rg1 in patients with carotid atherosclerosis and to define the scope of its clinical benefits.

## Data Availability

The original contributions presented in the study are included in the article/Supplementary Material, further inquiries can be directed to the corresponding authors.
