# Supplementary material for: The action of ginsenoside Rg1 in patients with carotid atherosclerosis: a controlled clinical trial
Source: Front Pharmacol. 2025 Sep 2;16:1638359. doi: 10.3389/fphar.2025.1638359 (PMC12436125; doi:10.3389/fphar.2025.1638359)
Supplement: Supplementary file 1 [file Supplementaryfile1.docx]

**The Action Mechanism of Ginsenoside Rg1 in Patients with Carotid Atherosclerosis: A Controlled Clinical Trial**

Xu Fang^a†^, Xuan Ma^cd†^, Man Zhang^a^, Li Zhang^a^, Xu He^a^, Shilei Liu^b^, Yinsong Dong^b^, Yan Li^a*^, Junzi Wu^cd*^

^a.^ Department of Geriatrics, the First People's Hospital of Yunnan Province, Kunming, Yunnan 650500, China;

^b.^ National-Local Joint Engineering Research Centre for Sanqi Resource Protection and Utilisation Technology, Kunming, Yunnan 650500, China;

^c.^ The Key Laboratory of Microcosmic Syndrome Differentiation, Yunnan University of Chinese Medicine, Kunming, Yunnan 650500, China;

^d.^ Yunnan Key Laboratory of Integrated Traditional Chinese and Western Medicine for Chronic Disease in Prevention and Treatment, Yunnan University of Chinese Medicine, Kunming, Yunnan 650500, China)

^†^These authors contributed equally to this work

^*^Corresponding authors at: Yan li, Junzi Wu, Yunnan University of Chinese Medicine, Kunming, Yunnan, China; E-mail addresses: liyanken@126.com (Y. Li); xnfz@ynucm.edu.cn (J. Wu).

Table S1

Table S1. Comparison of laboratory examination indicators among the three groups of patients

|  | Group 1（n=32） | Group 2（n=33） | Group 3（n=41） | P |
| --- | --- | --- | --- | --- |
| WBC(×109/L) | 6.210(5.2,6.6) | 5.320(4.9,6.5) | 5.405(4.4,6.5) | 0.316 |
| NEUT (%) | 55.950(49.7,62.8) | 54.600(50.6,61.3) | 57.550(51.1,60.1) | 0.873 |
| RBC(×1012/L) | 5.030(4.7,5.2) | 4.990(4.7,5.5) | 4.945(4.8,5.2) | 0.878 |
| HGB(g/L) | 150.500(144.3,159.8) | 149.000(143.5,162.0) | 151.000(143.3,157.0) | 0.974 |
| HCT | 0.462(0.4,0.5) | 0.458(0.4,0.5) | 0.458(0.4,0.5) | 0.955 |
| PLT(×109/L) | 235.56±48.64 | 227.18±53.76 | 230.03±43.86 | 0.794 |
| ALT(U/L) | 23.000(16.3,30.8) | 20.000(16.0,26.5) | 21.000(16.3,25.8) | 0.348 |
| AST(U/L) | 24.500(20.3,28.8) | 23.000(18.0,28.0) | 23.000(20.0,26.0) | 0.497 |
| TBIL(μmol/L) | 12.550(10.4,17.0) | 12.500(9.4,16.5) | 13.900(10.8,17.6) | 0.616 |
| TP（g/L） | 74.550(71.0,77.2) | 72.500(69.8,76.0) | 75.350(71.3,78.5) | 0.071 |
| ALB（g/L） | 45.80±2.79 | 45.15±2.11 | 45.98±1.96 | 0.224 |
| GLB g/L | 28.83±4.11 | 27.58±3.41 | 28.77±4.21 | 0.295 |
| Blood lipids | | | | |
| TG (mmol/L) | 1.670(1.3,2.2) | 1.600(1.0,2.1) | 1.560(1.1,2.0) | 0.701 |
| TC（mmol/L） | 5.670(1.3,6.2) | 5.600(1.0,6.1) | 5.560(1.1,6.0) | 0.631 |
| HDL-C(mmol/L) | 1.270(1.1,1.5) | 1.330(1.2,1.7) | 1.430(1.2,1.6) | 0.222 |
| LDL-C(mmol/L) | 3.010(2.3,3.6) | 2.955(2.0,3.5) | 3.300(2.3,4.0) | 0.330 |

Table S1

Table S1. Comparison of laboratory examination indicators among the three groups of patients(continued)

| Serum pro-inflammatory mediators | | | | |
| --- | --- | --- | --- | --- |
| IL-10（pg/ml） | 3.400(1.9,6.5) | 2.860(2.4,5.0) | 3.050(2.6,4.3) | 0.815 |
| IL-2（pg/ml） | 1.570(0.8,4.3) | 1.700(1.1,3.1) | 1.240(0.6,3.3) | 0.717 |
| IL-4（pg/ml） | 1.870(1.0,4.3) | 1.690(0.9,2.8) | 2.180(1.0,3.5) | 0.647 |
| IL-6（pg/ml） | 6.630(3.1,9.9) | 6.640(2.3,12.2) | 5.720(3.4,11.5) | 0.984 |
| TNF-α (μg/L) | 1.876(0.9,4.7) | 1.560(1.5,2.5) | 1.710(1.0,2.9) | 0.763 |
| IFN-r(μg/L) | 5.006(2.7,9.3) | 3.920(2.3,6.7) | 4.422(2.8,6.8) | 0.532 |
|  |  | Other |  |  |
| HCY（umol/L） | 13.700(11.2,16.8) | 13.400(10.5,18.0) | 13.300(11.4,15.4) | 0.862 |
| 25（OH）D | 23.370(18.8,29.7) | 23.640(19.7,31.9) | 26.220(21.9,30.9) | 0.056 |
| IR | 2.672(1.3,4.1) | 2.105(1.3,3.8) | 2.537(1.1,3.7) | 0.274 |
| HbA1C | 6.010(5.9,6.2) | 6.060(5.8,6.7) | 5.870(5.7,6.2) | 0.140 |

Table S2

Table S2. Comparison of antioxidant indexes among the three groups

|  | Group1（n=32） | Group2（n=33） | Group3（n=41） | P |
| --- | --- | --- | --- | --- |
| MDA | 9.068(8.7,9.7) | 8.622(8.3,9.4) | 8.915(8.5,9.6) | 0.304 |
| ROS | 8.298(7.9,8.7) | 8.255(7.7,8.9) | 8.392(7.9,8.8) | 0.925 |
| SOD | 207.956(196.9,223.9) | 213.265(201.7,233.8) | 204.638(195.4,227.8) | 0.207 |
| GSH | 81.277(75.5,87.2) | 81.415(75.1,87.3) | 78.426(74.5,82.6) | 0.491 |

Table S3

Table S3. Comparison of vascular aging indexes among three groups

|  | Group1（n=32） | Group2（n=33） | Group3（n=41） | P |
| --- | --- | --- | --- | --- |
| Lumica-n | 31.550(30.4,34.4) | 31.770(30.4,33.6) | 31.450(30.1,34.0) | 0.865 |
| FGF21 | 1305.730(1228.1,1432.3) | 1300.950(1211.5,1411.3) | 1311.500(1213.6,1412.1) | 0.941 |
| Fibulin1 | 318.890(301.4,345.5) | 333.190(312.1,359.6) | 328.600(315.3,348.6) | 0.913 |

Table S4

Table S4. Comparison of atherosclerosis indexes in three groups

|  | Group1（n=32） | Group2（n=33） | Group3（n=41） | P |
| --- | --- | --- | --- | --- |
| Right Ankle-Brachial Index (ABI) | 1.160(1.1,1.3) | 1.198(1.1,1.3) | 1.237(1.1,1.3) | 0.968 |
| Right Ankle-Brachial PWV | 1621.00  (1502.0,1735.0) | 1691.00  (1446.0,1958.5) | 1567.00  (1402.5,1725.5) | 0.210 |
| Left Ankle-Brachial Index (ABI) | 1.178(1.1,1.3) | 1.151(1.1,1.3) | 1.193(1.1,1.3) | 0.974 |
| Left Ankle-Brachial PWV | 1695.61±221.87 | 1796.33±323.81 | 1691.90±301.75 | 0.289 |

Table S5

Table S5. Comparison of plaque locations before treatment between the three groups

| Plaque location (number) | amount（n=162） | Group1（n=53） | Group2（n=51） | Group3（n=57） | P |
| --- | --- | --- | --- | --- | --- |
| common carotid artery | | | | | |
| Left | 45（27.78%） | 12（22.64%） | 14（27.45%） | 19（33.33%） | 0.885 |
| Right | 46（28.40%） | 17（32.07%） | 15（29.42%） | 14（24.56%） |  |
| subclavian artery | | | | | |
| Left | 0 | 0 | 0 | 0 | 8.625 |
| Right | 47（29.01%） | 16（30.19%） | 17（33.33%） | 14（24.56%） |  |
| internal carotid artery | | | | | |
| Left | 11（6.79%） | 3（5.66%） | 4（7.84%） | 4（7.02%） | 0.462 |
| Right | 11（6.79%） | 4（7.55%） | 1（1.96%） | 6（10.53%） |  |
| external carotid artery | | | | | |
| Left | 1（0.62%） | 1（1.89%） | 0 | 0 | 1.000 |
| Right | 1（0.62%） | 1（3.23%） | 0 | 0 |  |

Table S6

Table S6. Comparison of scale scores among three groups of patients

|  | Group1（n=32） | Group2（n=33） | Group3（n=41） | P |
| --- | --- | --- | --- | --- |
| MOCA | 24.000(22.0,27.0) | 25.000(23.0,26.0) | 23.000(21.5,26.0) | 0.278 |
| HAMA | 6.000(5.0,8.0) | 6.000(2.0,8.0) | 6.000(2.0,8.5) | 0.171 |
| PSQI | 5.000(3.5,6.0) | 5.000(4.0,7.5) | 6.000(4.0,7.4) | 0.431 |
| HAMD | 6.000(5.0,11.0) | 6.000(4.5,9.0) | 6.000(4.0,8.5) | 0.616 |

Figure S1


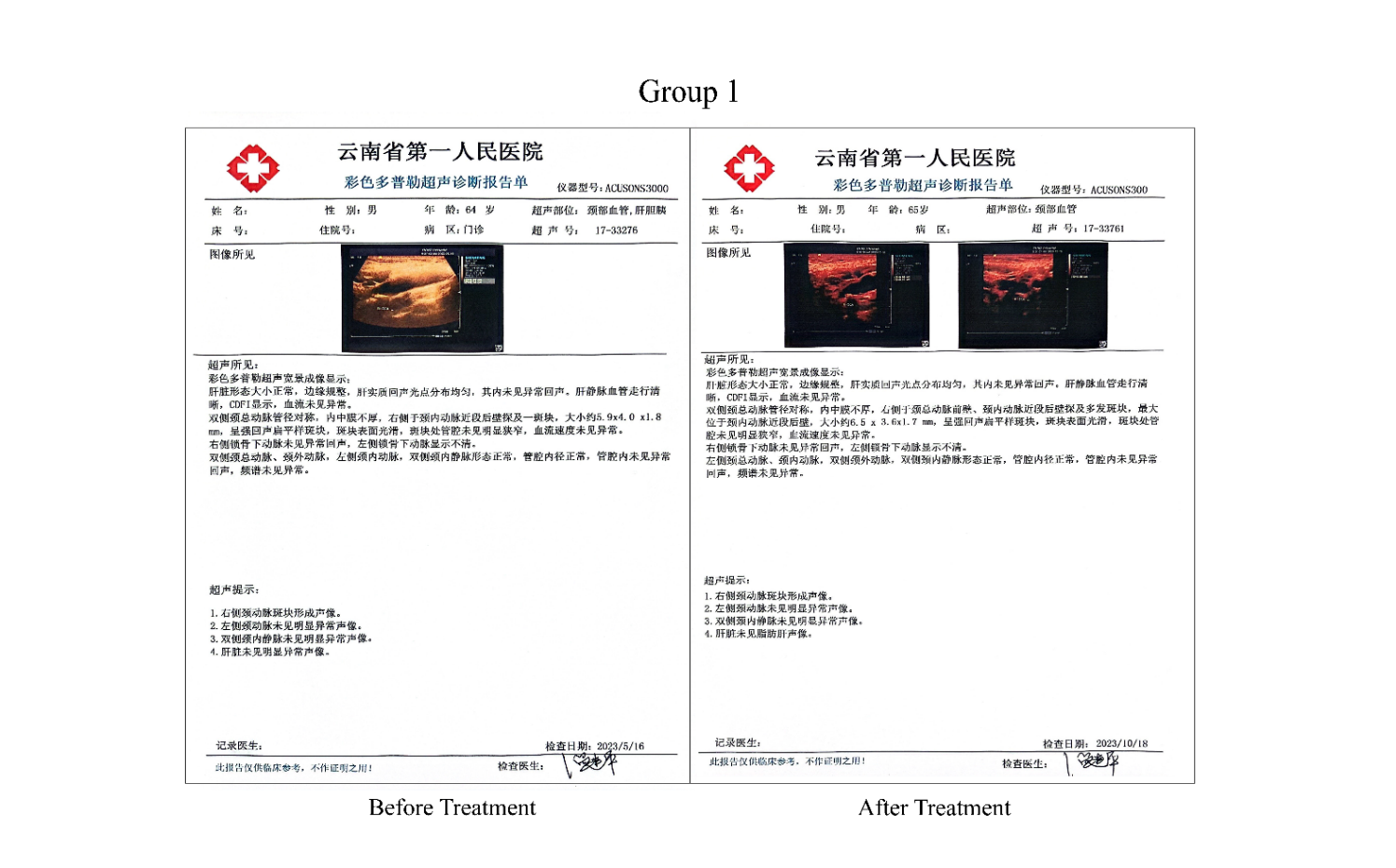


Figure S1 Group 1. received lifestyle interventions plus atorvastatin calcium tablets (n = 32).

Note: (1) Before Treatment: On the right side, a plaque is identified on the posterior wall of the proximal internal carotid artery, measuring approximately 5.9 × 4.0 × 1.8 mm. (2) After Treatment: Multiple plaques are detected on right common carotid artery anterior wall and internal carotid artery posterior wall of the proximal segment. The largest plaque, measuring approximately 6.5×3.6×1.7 mm, is located on the posterior wall of the proximal internal carotid artery.

Figure S2


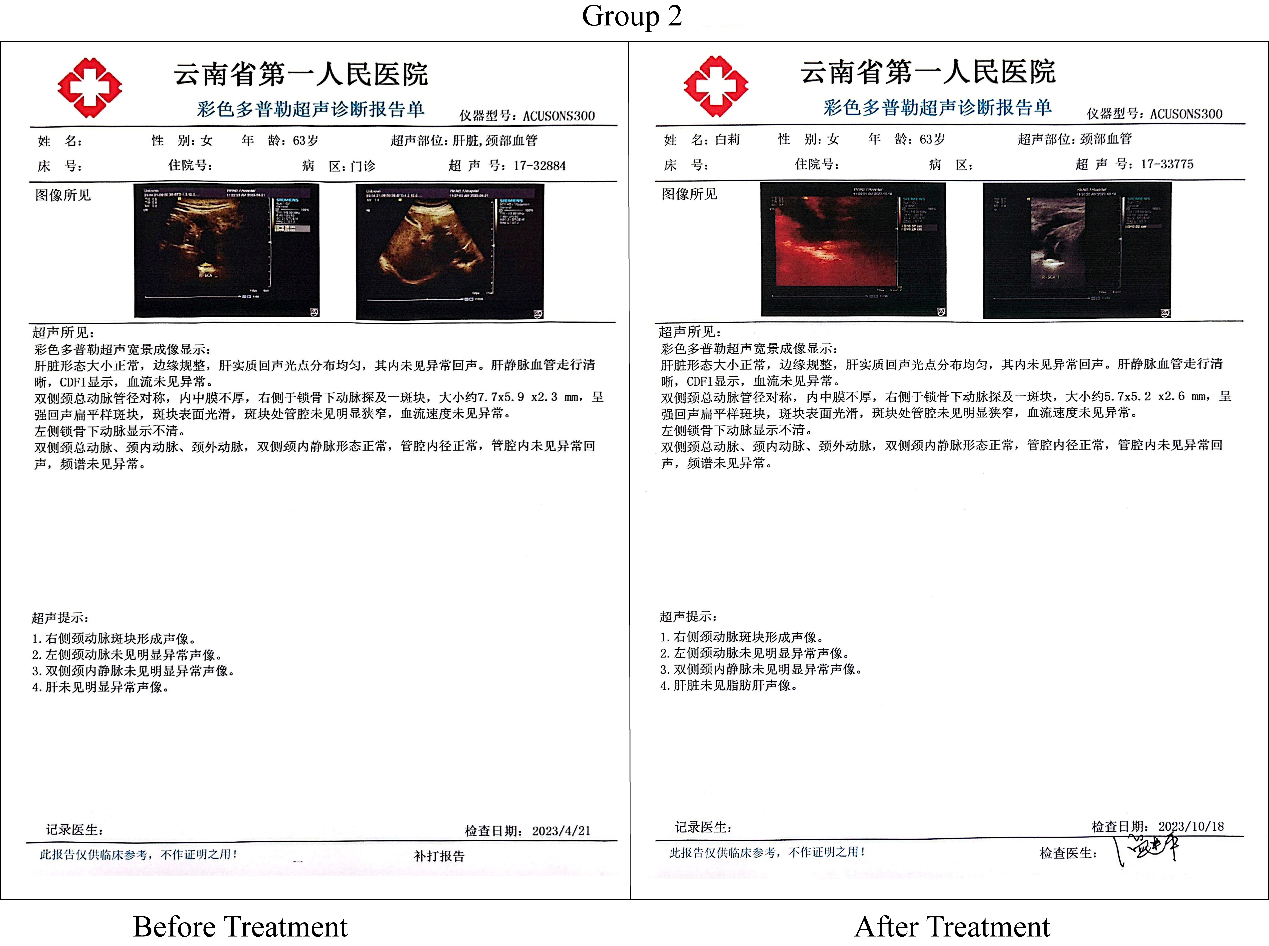


Figure S2 Group 2. received the same treatment as Group 1, with the addition of Bayaspirin (n = 33).

Note: (1) Before Treatment: A plaque is identified in the right subclavian artery, measuring 7.7 × 5.9 × 2.3 mm. The plaque appears as a hyperechoic, flat lesion with a smooth surface. (2) After Treatment: A plaque is identified in the right subclavian artery, measuring 5.7 × 5.2 × 2.6 mm. The plaque appears as a hyperechoic, flat lesion with a smooth surface.

Figure S3


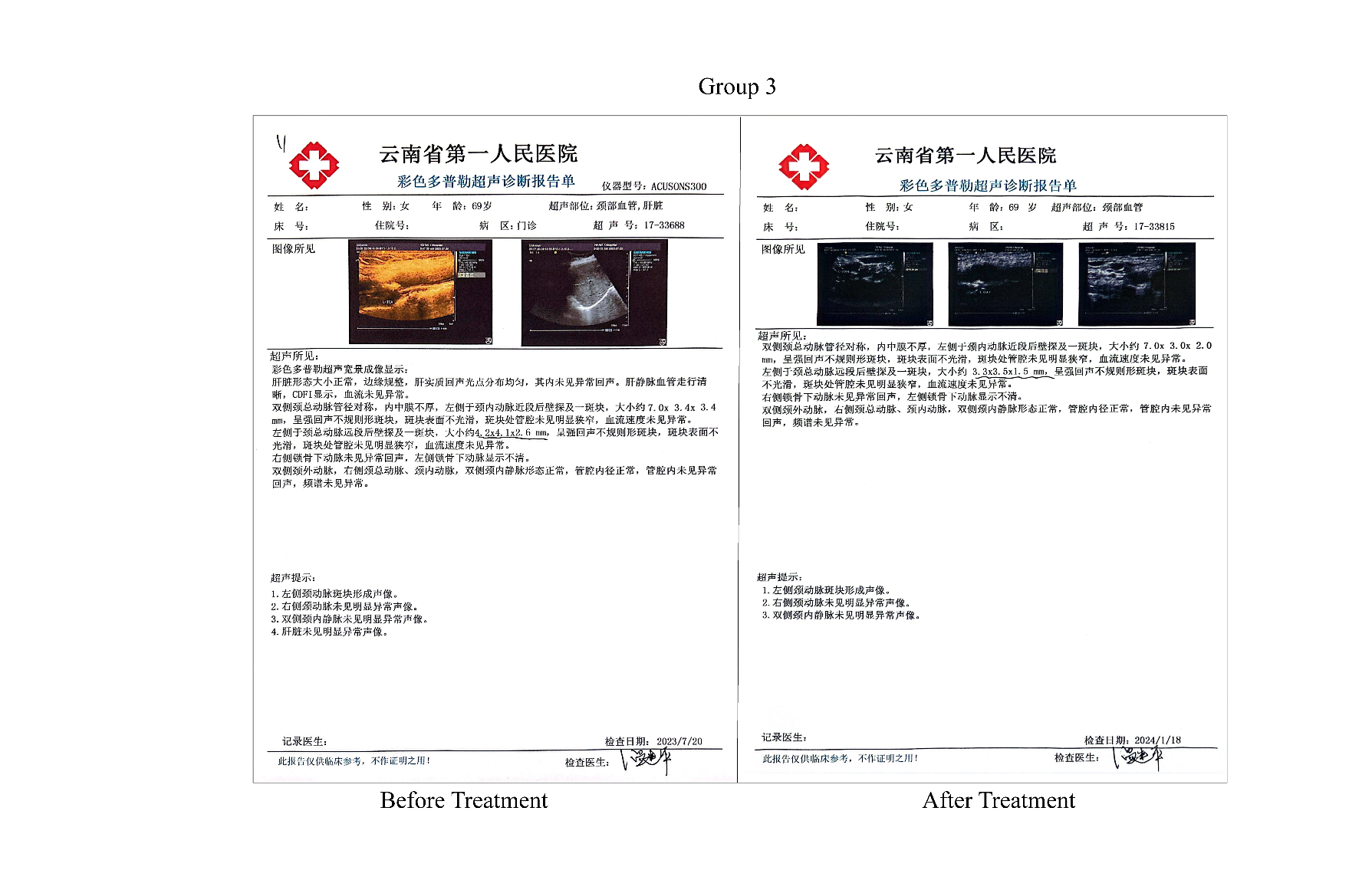


Figure S3 Group 3. received the same treatment as Group 2, but with Bayaspirin replaced by ginsenoside Rg1 (n = 41).

Note: (1) Before Treatment: On the left side, a plaque is detected in internal carotid artery proximal segment posterior wall, measuring about 7.0*3.4*3.4 mm, with a strongly echogenic irregularly shaped plaque, the surface of the plaque not being smooth, without causing significant luminal stenosis. Meanwhile, a plaque is detected in the posterior wall of the distal common carotid artery, measuring about 4.2*4.1*2.6 mm, with a strongly echogenic irregularly shaped plaque, and the surface of the plaque not being smooth. (2) After Treatment: On the left side, a plaque is detected in internal carotid artery proximal segment posterior wall, measuring about 7.0*3.0*2.0 mm, with a strongly echogenic irregularly shaped plaque, the surface of the plaque not being smooth, without causing significant luminal stenosis. Meanwhile, a plaque is detected in the posterior wall of the distal common carotid artery, measuring about 3.3*3.5*1.5 mm, with a strongly echogenic irregularly shaped plaque, and the surface of the plaque not being smooth.
